# Reduced visual evoked potential amplitude in autism spectrum disorder, a variability effect?

**DOI:** 10.1038/s41398-019-0672-6

**Published:** 2019-12-18

**Authors:** Klara Kovarski, Joëlle Malvy, Raoul K. Khanna, Sophie Arsène, Magali Batty, Marianne Latinus

**Affiliations:** 1 UMR 1253, iBrain, Université de Tours, Inserm, Tours, France; 2 CNRS (Integrative Neuroscience and Cognition Center, UMR 8002), Paris, France; 3 Université Paris Descartes, Sorbonne Paris Cité, Paris, France; 4 Fondation Ophtalmologique A. de Rothschild, Paris, France; 5 CHRU de Tours, Centre Universitaire de Pédopsychiatrie, Tours, France; 6 CHRU de Tours, Département d’Ophtalmologie, Tours, France; 7 Université de Toulouse, CERPPS, Toulouse, France

**Keywords:** Neuroscience, Autism spectrum disorders, Neuroscience, Autism spectrum disorders

## Abstract

Atypical sensory behaviours represent a core symptom of autism spectrum disorder (ASD). Investigating early visual processing is crucial to deepen our understanding of higher-level processes. Visual evoked potentials (VEPs) to pattern-reversal checkerboards were recorded in ASD children and age-matched controls. Peak analysis of the P100 component and two types of single-trial analyses were carried out. P100 amplitude was reduced in the ASD group, consistent with previous reports. The analysis of the proportion of trials with a positive activity in the latency range of the P100, measuring inter-trial (in)consistency, allowed identifying two subgroups of ASD participants: the first group, as control children, showed a high inter-trial consistency, whereas the other group showed an inter-trial inconsistency. Analysis of median absolute deviation of single-trial P100 (st-P100) latencies revealed an increased latency variability in the ASD group. Both single-trial analyses revealed increased variability in a subset of children with ASD. To control for this variability, VEPs were reconstructed by including only positive trials or trials with homogeneous st-P100 latencies. These control analyses abolished group differences, confirming that the reduced P100 amplitude results from increased inter-trial variability in ASD. This increased variability in ASD supports the neural noise theory. The existence of subgroups in ASD suggests that the neural response variability is not a genuine characteristic of the entire autistic spectrum, but rather characterized subgroups of children. Exploring the relationship between sensory responsiveness and inter-trial variability could provide more precise bioclinical profiles in children with ASD, and complete the functional diagnostic crucial for the development of individualized therapeutical projects.

## Introduction

Autism spectrum disorder (ASD) is characterized by (i) disturbances in the social and communication domain, and by (ii) repetitive and restricted behaviours. Within this second group of symptoms, sensory abnormalities, as acknowledged in the Diagnostic and Statistical Manual-5 (DSM-5) are now considered a diagnostic feature of ASD^1^. These might be defined as hypo- or hyper-responsiveness in all sensory modalities, making atypical sensory behaviours highly heterogeneous both within and between autistic individuals. In the visual modality, children with ASD might be fascinated by lights, they might stimulate their eyes with their hands or might inspect objects in atypical manners^2^. In parallel, autistic individuals might also avoid challenging stimuli, such as others’ gaze or scenes rich in information. In line with this, an increasing number of investigations using electroencephalography (EEG) have revealed that visual abnormalities are found when social but also non-social stimuli are presented: from atypical evoked-related potentials (ERPs) to atypical frequency-band responses, to stimuli such as faces, gabors and gratings^3–9^. Atypical brain responses are additionally supported by structural abnormalities in visual regions^10^, as revealed by different size and organization of micro-columns in those with ASD compared with controls^11^. Taken together, these findings have been acknowledged by recent reviews, suggesting that investigating early sensory responses might provide crucial information to understand higher-level disturbances^12,13^.

Among the different visual evoked potentials (VEPs) used for testing the integrity of the visual pathways, pattern-reversal checkerboards VEPs provide highly reliable waveforms. Accordingly, this visual response, recorded over Oz, represents a clinical tool to test the functional integrity of the visual system at the individual level^14^. In a previous study using pattern-reversal checkerboards VEPs, we showed that P100 amplitude was decreased in adolescents and young adults with ASD^7^. However, as ASD is a neurodevelopmental condition, investigating brain responses in young children is necessary to understand the developmental trajectory of this condition and to determine whether this reduced response is a genuine feature of ASD. More precisely, if a reduced P100 amplitude is also found in younger subjects, this leads to conclude that this atypicality is an early feature of autism. However, the reduced P100 in adults could result from a progressive modification of sensory processing in childhood as a consequence of the difficulty to deal with sensory information in everyday life; under this assumption, no reduction of amplitude would be observed in young children. This is particularly important as previous studies of VEPs with gratings in school-aged children and adolescent did not report differences in VEPs between typically developing (TD) and ASD children^15^, which could suggest that VEPs alter with age.

Easy to record in young participants, these VEPs represent an ideal tool to evaluate early stages of visual processing in those with ASD, especially at the low-end of the spectrum, often neglected in research studies^16–18^. Although ERPs might be easily computed, they reflect an average response that masks inter-trial variability, known to be an important feature of ASD^15,19–21^ and in other neurodevelopmental disorders such as Attention Deficit Hyperactivity Disorder (ADHD)^6,15,19,22,23^. In ASD, this increased variability has been explained by an increased neural noise, as described by the neural noise theory^15,24–27^ (but see ref. ^28^). This account interprets response variability reported in neuroimaging studies as a signature of abnormalities in the synchronization of neural activity, which might be crucial in ASD^27^. Accordingly, single-trial analyses of EEG data represent a useful tool in order to assess neural response variability. However, only few previous studies have investigated neural responses variability in children with ADHD^22^ and in school-aged children and adolescents with ASD^15,20^.

Although response variability has been previously described in school-age children and adolescents with autism^15^, here we deepen our understanding by linking differences in VEPs to inter-trial variability^7^. In addition, investigating this variability of visual responses in children with ASD might help provide further information on the developmental aspects of early visual impairments, but also on the compensatory mechanisms that might develop with age^29^.

## Materials and methods

### Participants

Children were recruited from the Child Psychiatry Department of the University Hospital of Tours, France. The initial ASD group included 29 children diagnosed with ASD by an experienced clinical team according to DSM-5 criteria^1,30^, and Autism Diagnostic Interview-Revised and/or the Autism Diagnostic Observation Schedule-generic assessments^31,32^. Epilepsy was the exclusion criterion. The ASD group received additional comprehensive ophthalmological and orthoptic examinations. These included tests for visual acuity refractive errors measurements under cycloplegia, as well as a slit-lamp and fundus examination, and an oculomotor exam. Seven children diagnosed with an ophthalmological disorder that could affect VEP responses were not included in the analysis (i.e., refractive errors, strabismus and amblyopia). Three other children were not included in the experiment, because they could not tolerate the electrodes cap, and the recording of one child was excluded because of an insufficient number of trials after artifacts rejection. The final group included 18 children aged 42 to 130 months (mean age ± SD: 89 ± 32 months; 1 girl and 17 boys; Childhood Autism Rating Scale scores from 24.5 to 38, mean: 30 ± 4.4)^33^. The ASD group was matched for chronological age to a group of eighteen TD children aged 37 to 143 months (mean age: 90 ± 31 months, *t* (34) = −0.14, *P* *=* 0.89; 3 girls and 15 boys). The local ethics committee approved the study (Clinical Trial: NCT02444117) and informed written consent was obtained from the parents of the children. The study was conducted according to the ethical principles of the Declaration of Helsinki.

### Procedure

Participants were seated in a chair 110 cm away from the screen and were presented with black and white pattern-reversal stimulations (1 Hz frequency presentation) for a maximum of 2 min. The stimulus consisted in 11 × 11 checks that fitted a screen of 19.8° (wide) × 15.6° (high) visual angle. Each check measured 1.8° x 1.4° visual angle.

### EEG recording and processing

A 64-channel ActiveTwo system (Biosemi^®^, The Netherlands) was used for EEG recording. Blinks and saccades were monitored using electrodes placed at the outer canthi of the eyes and below the left eye. For offline reference an electrode was applied to the tip of the nose. The signal was recorded at a sampling frequency of 512 Hz and filtered at 0–104 Hz. A 0.3 Hz digital high-pass filter was applied to the EEG signal. Ocular artifacts were corrected by applying independent component analysis (ICA) as implemented in EEGLab^34^, Matlab, The Mathworks, Inc. Blink artifacts were captured into components that were automatically removed via the inverse ICA transformation. Other artifacts (i.e., muscle and movements) were rejected manually with the Elan software^35^. Continuous EEG signal was time-locked to trial onset and trials were extracted between −100 ms pre-stimulus and 400 ms post-stimulus. Baseline correction (−100 to 0 ms) was applied and ERPs were digitally filtered with a low-pass frequency cut-off of 30 Hz.

Data were re-referenced offline to the potential of the tip of the nose. The number of trials averaged to compute ERPs was 110.4 ± 30.8 in ASD children and 99.5 ± 16.8 in TD children; the groups did not differ (*t*(34) = 1.32, *P* *=* 0.19; see ref. ^14^). Single trials were extracted with Matlab from the preprocessed data (all steps but baseline correction and low-pass filtering). Baseline correction was performed on the 100 ms preceding stimulus onset and a low-pass filter (cut-off frequency: 30 Hz) was applied on each trial using an EEGLab function (eegfilt with a Finite Impulse Response).

### Statistical analysis

Three occipital electrodes were selected for the analyses: O1, Oz and O2. These electrodes were selected based on recommendations regarding analysis of pattern-reversal VEPs^14^ and on previous studies reporting hemispherical differences between ASD and TD individuals^3^.

Two analyses were carried out: the first was based on the usual peak latency and amplitude (ERPs analysis), whereas the second aimed at considering intra-participant variability by looking at inter-trial variability (single-trial analyses).

### ERPs analysis

P100 was defined as the greatest positive deflection in the 70–140 ms time window. Two analyses of variance (repeated-measure analysis of variance (ANOVA); one for amplitude and one for latency) were carried out on the P100 with Group (ASD and TD) as a between-subject factor and Electrodes (O1, Oz and O2) as a within-subject factor. Data met ANOVA assumptions: normality of residuals was assessed by visual inspection of a *q*–*q* plot in Statistica and a Shapiro–Wilk’s test (*W* > 0.94; *P* > 0.09); variance was homogeneous across groups for the within-subject factor (Levene’s test: all F(1,34) > 3.5; all *P*s > 0.075). For all ANOVAs performed, Bonferroni post-hoc corrections were applied where needed. *F* and corrected *P*-values are provided, as well as effect sizes (partial eta squared ${\upeta}_{\mathrm{p}}^2$). Significance was considered for *P* < 0.05.

### Single-trial analyses

First, for each participant, we measured the proportion of trials showing a positive activity at each time point for each of the three electrodes. Significance was assessed by means of bootstrapping with replacement (5000 bootstraps) of all trials in a single subject, and calculating the proportion of trials with a positive activity after each bootstrap. A 95% confidence interval was then estimated around the ‘true’ proportion of trials showing a positive activity. A subject was deemed as having a significant proportion of trials with a negative/positive activity if the confidence interval did not include 50% (e.g., the proportion obtained by chance) on 5 consecutive time points on the three electrodes of interest between 70 and 140 ms (for a similar analysis, see ref. ^20^).

It has been previously shown that trial-by-trial variability of P100 latency and amplitude was increased in those with ASD^15^. Accordingly, in the current study additional analyses were performed on the single-trial P100 (st-P100) component (latency and amplitude) to investigate the influence of trial-by-trial variability on the ERPs^36^. Single-trial peak analysis was performed with Matlab. st-P100 was considered as the maximum peak in the 70–140 ms time window, closest to the latency of the individual P100 component if several peaks were identified within this time window. If no peak was identified, the peak was measured as the maximal value in the predefined time window for the P100. Mean latencies and amplitudes (including all trials) of the components were measured for each participant and repeated-measure ANOVAs were performed for both components with Group (ASD and TD) as a between-subject factor and Electrodes (O1, Oz and O2) as a within-subject factor. To evaluate inter-trial variability in latency and amplitude, the median absolute deviation (MAD) was computed for each subject at each electrode (for a similar analysis see^15,22^).

## Results

### ERPs results

**Fig. 1:**
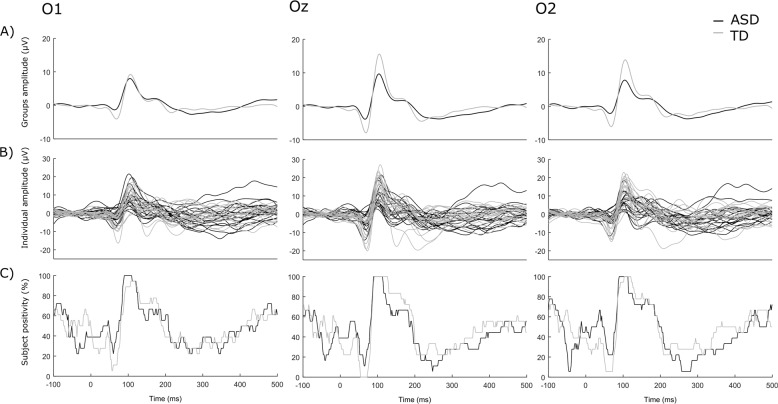
VEPs over the three electrodes of interest in the ASD (black line) and the TD groups (grey line). **a** Grand average of both groups (dark: ASD; light: TD). Note that on O2, P100 amplitude was 8.3 µV ± 4.3 for children with ASD and 14.3 µV ± 6.3 for TD children. **b** Individual data. **c** Percentage of participants presenting positive VEPs at each time point.

Grand average responses over the three electrodes of interest are shown in Fig. 1a, whereas individual VEPs for both groups are shown in Fig. 1b.

Regarding P100 latency, a significant effect of Electrode was found (F(1.62,55.2) = 5.14, *P* *=* 0.013, ${\upeta}_{\mathrm{p}}^2$ = 0.131), due to latency being shorter over Oz than O2 (*P* = 0.037) and O1 (*P* = 0.037). There was neither an effect of Group, nor an Electrode by Group interaction on P100 latency (*P* > 0.20).

On P100 amplitude, main effects of Group (F(1,34) = 6.00, *P* = 0.019, ${\upeta}_{\mathrm{p}}^2$ = 0.15) and Electrodes (F(1.67,56.8) = 23.79, *P* *<* 0.001, ${\upeta}_{\mathrm{p}}^2$ = 0.412) were observed, further characterized by a Group by Electrodes interaction (F(1.67,56.8) = 12.1, *P* *<* 0.001, ${\upeta}_{\mathrm{p}}^2$ = 0.263). Planned comparisons for the interaction (within-group across electrodes and between-groups at each electrode; *n* = 9) revealed a smaller amplitude in the ASD group as compared with the TD group on O2 (*P* = 0.029) and Oz (*P* = 0.023) but not on O1 (*P* > 0.5), and that only the TD group presented greater amplitude on O2 and Oz compared with O1 (both *P* < 0.001).

Inter-subject variability, measured as the proportion of participants of each group showing a positive activity around the P100 (Fig. 1c), highlighted that all participants of both groups showed a positive deflection on their averaged ERPs between 90 and 100 ms, suggesting that the differences observed on the P100 are not driven by the absence of a positive response on the ERPs in some participants.

To provide further understanding of these differences we investigated intra-subject variability by performing single-trial analyses.

### Single-trial results

#### Inter-trial consistency

**Fig. 2:**
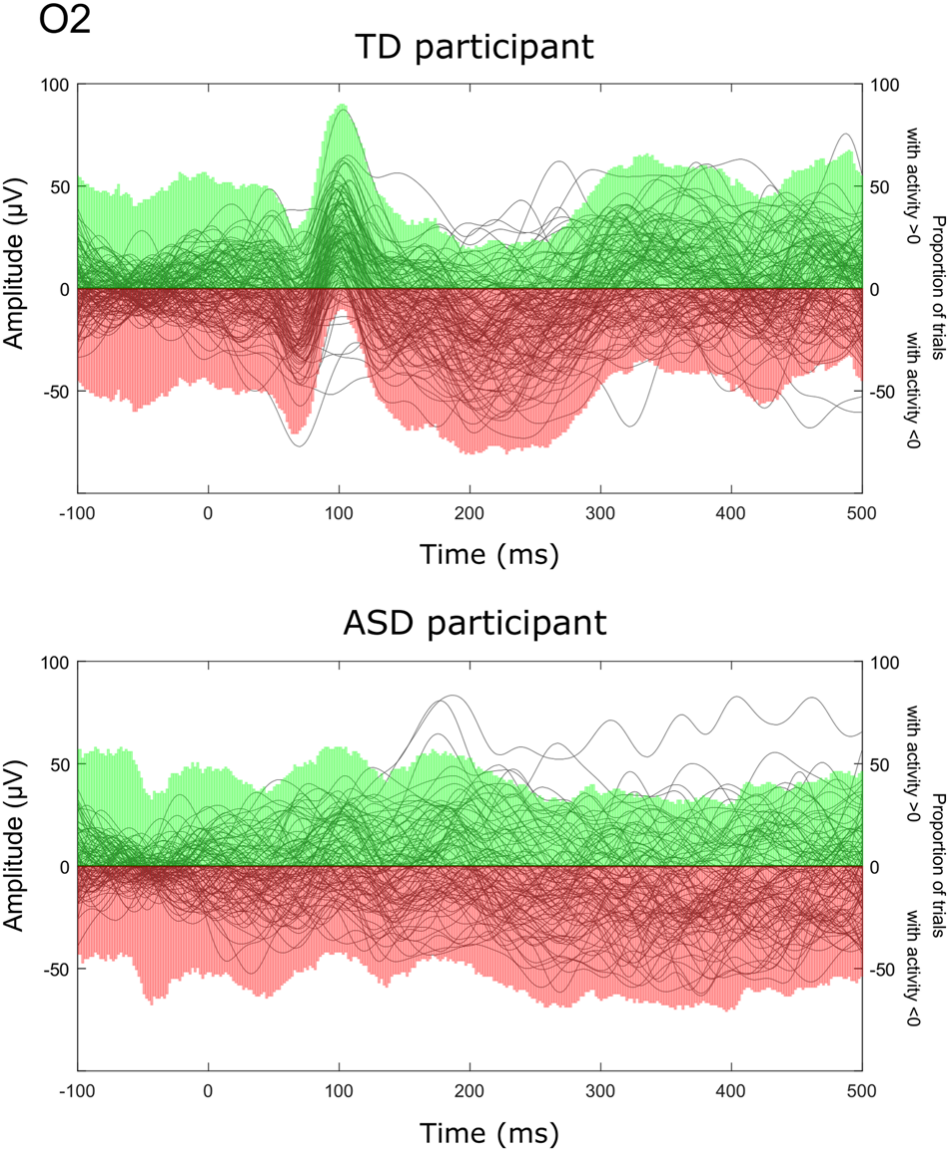
Individual examples for a TD participant (top) and an ASD participant (bottom) showing the percentage of trials showing a positive deflection at each time point on O2. Green colour indicates the percentage of trials with positive activity, whereas red colour indicates (minus) the percentage of trials with negative activity. Note that the length of each ‘bicolour’ bar is therefore 100%.

**Fig. 3:**
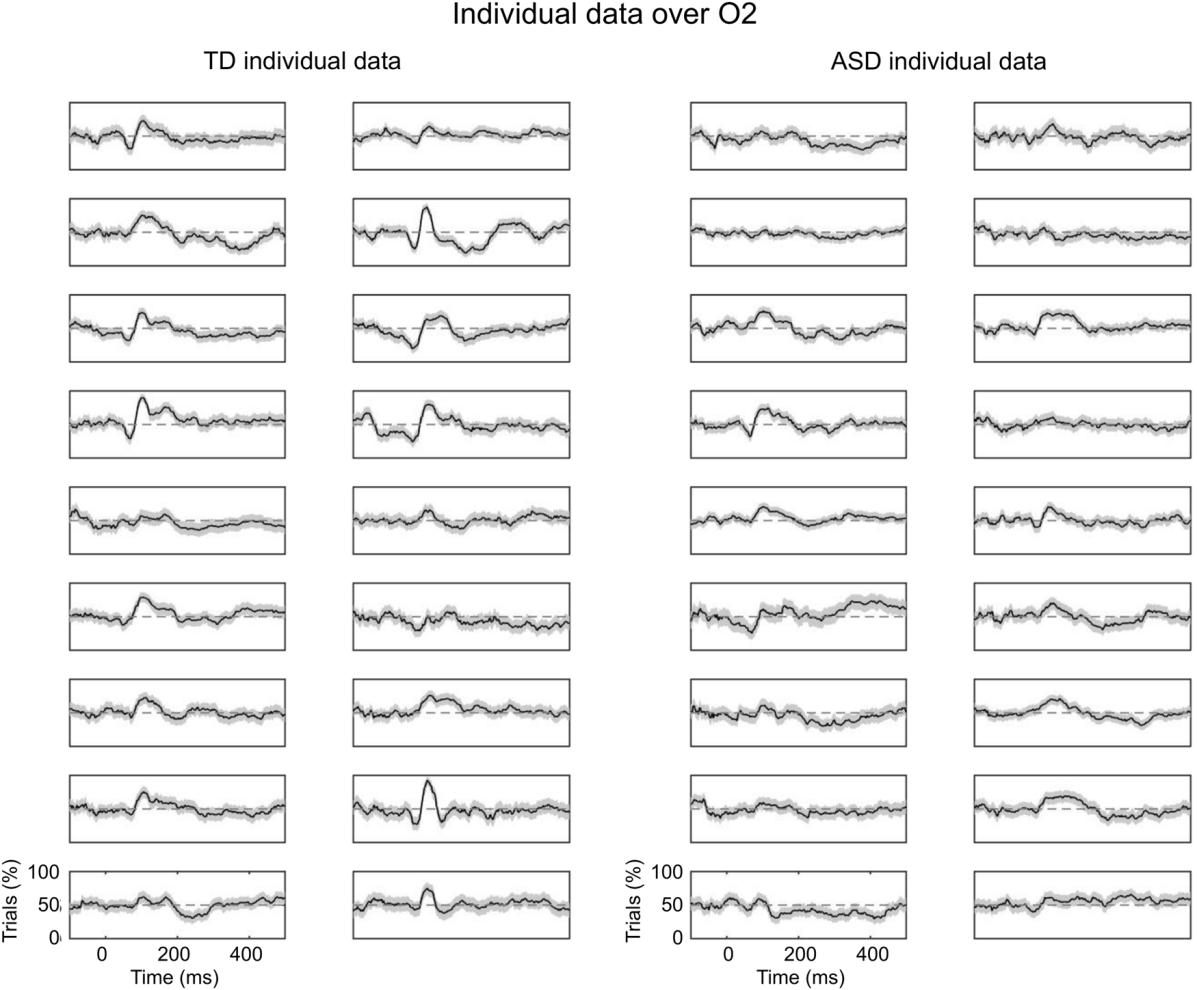
Individual data of inter-trial consistency illustrated on O2. Left side: proportion of trials with a positive activity in all TD children. Right side: proportion of trials with a positive activity in all ASD children. Shaded area represent 95% confidence intervals around the proportion of positive trials.

Figure 2 illustrates this single-trial approach for one TD and one ASD child. The maximal proportion of positive trials observed on Oz in the TD group was 94% and 79% in children with ASD (Fig. 3). A proportion of trials with positive activity significantly greater than chance in the latency range of the P100 was observed in 78% of TD participants and 44% of children with ASD. This analysis allowed identifying two subgroups of ASD participants: in the first group (*n* = 8), there was a significant proportion of trials with positive amplitude reflecting a high inter-trial consistency (P100 amplitude, on O2, was 11.7 µV ± 3.2), whereas the other subgroup (*n* = 10) was characterized by a nonsignificant proportion of positive trials traducing a low inter-trial consistency (P100 amplitude on O2 was 5.6 µV ± 2.9).

#### Peak variability

The ANOVA performed on the mean amplitude of the st-P100 revealed a Group by Electrodes interaction (F(2,68) = 9.85, *P* *<* 0.001, ${\upeta}_{\mathrm{p}}^2$ = 0.225); yet, planned comparisons revealed no between group differences (all corrected *P*s > 0.16). It is noteworthy that in the TD group, the effect of electrodes remained (all corrected *P*s < 0.001). On O2, mean st-P100 amplitude was 15.3 ± 4.1 in ASD and 19.7 µV ± 5.9 in TD.

We then measured the MAD of st-P100 amplitudes and latencies in each child.

Although no Group effect was found on the MAD of st-P100 amplitudes, a main effect of Electrodes, further characterized by an interaction between Group and Electrodes, was observed (F(1.56,52.99) = 6.38, *P* = 0.006, ${\upeta}_{\mathrm{p}}^2$ = 0.157); planned comparisons revealed no relevant between-groups differences, but MAD was greater on O2 and Oz than on O1 in the TD group (corrected *P* < 0.005).

The MAD of st-P100 latencies was significantly larger for the ASD group than the TD group (F(1,34) = 7.73, *P* = 0.0088, ${\upeta}_{\mathrm{p}}^2$ = 0.18). Electrodes also affected the MAD of latencies (F(1.58,53.9) = 19.9, *P* < 0.001, ${\upeta}_{\mathrm{p}}^2$ = 0.37), as it was the smallest on Oz and the largest on O1; all pairwise comparisons were significant (Bonferroni corrected).

MAD of st-P100 latencies was within 1 SD from the mean for all three electrodes in 89% of the TD children and in 44% of the subjects with ASD. Based on the MAD of st-P100 latencies, two subgroups of ASD participants can also be identified: in the first group (*n* = 8) the MAD was within 1 SD (P100 amplitude, on O2, was 10.9 µV ± 3.9); in the second group the MAD (*n* = 10) was larger than 1 SD (P100 amplitude, on O2, was 6.3 µV ± 3.6).

By measuring st-P100 amplitudes, we showed that the group difference was no longer significant, suggesting that the single-trial approach leads to different results as compared to ERPs. The latency MAD analysis revealed that children with ASD presented a larger inter-trial latency variability than TD children. VEPs result from an averaging procedure strongly relying on the assumption of inter-trial consistency, i.e., an ERP is elicited at roughly the same latency for each trial. A larger trial-to-trial latency variability has the potential to blur the average VEP waveform. To go further in our understanding, we tested whether controlling inter-trial variability allows reducing group differences.

### Controlling analyses

#### Controlling amplitude variability at each time point

To control the effect of inter-trial inconsistency, we reconstructed ERPs with only trials with a positive response on five consecutive time points in the latency range of the P100 (i.e., between 70 and 140 ms) and on the three electrodes of interest. The reconstructed ERPs included on average 73 trials for the children with ASD (range: [33 121]) and 70 for the TD children (range: [36 104]). An ANOVA on amplitude of reconstructed P100 revealed a main effect of electrodes and a Group by Electrodes interaction (F(2,68) = 12.75, *P* < 0.001, ${\upeta}_{\mathrm{p}}^2$ = 0.27) as TD children presented a larger P100 on O2 and Oz. It is noteworthy that the main effect of group and the group differences on O2 and Oz observed on the original ERPs were no longer significant.

#### Controlling for latency variability

**Fig. 4:**
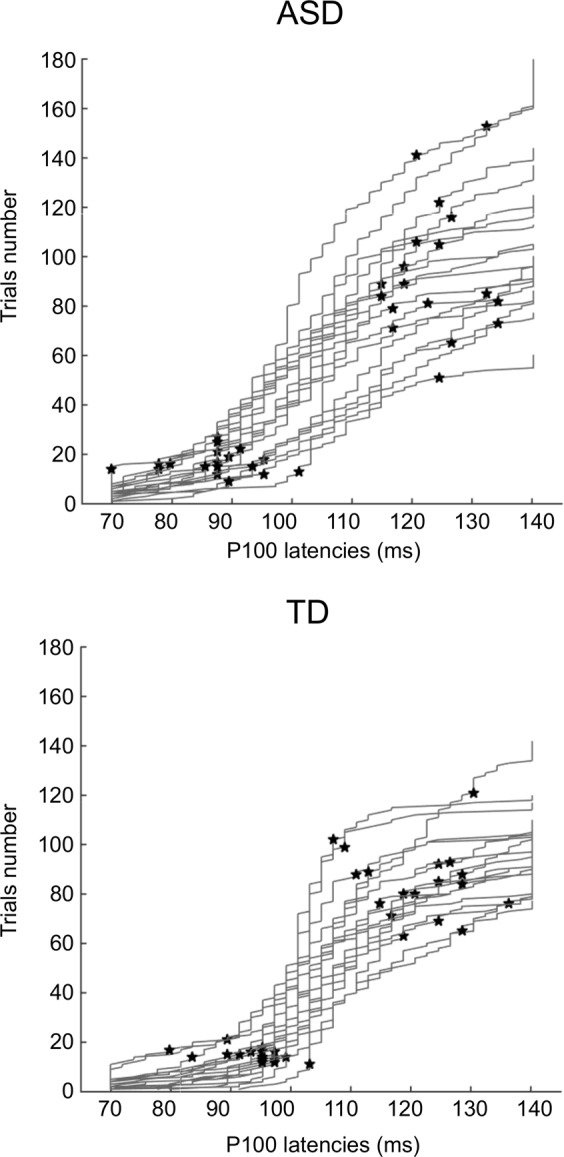
Illustration of single-trial P100 latencies in each individual for TD and ASD groups. Latencies are sorted from the smallest to the largest. A line represents single-trial latencies measured for one participant. The black stars mark the limit of the latencies used in the analysis of homogenous latencies. [Note the larger variability of single-trial latencies in ASD participants seen in less steep slopes.].

To control for latency variability, we rebuilt the trimmed mean ERPs by selecting only trials that presented a homogeneous P100 latency. This analysis allows reducing the influence of the tails of a distribution by removing them^37^. Practically, for each electrode, the 15% of trials that had the earliest latency and the 15% that had the longest latency were excluded from the ERPs generation (see Fig. 4). Amplitude and latency of the P100 component were then measured on the reconstructed ERPs (*N* trials [Range]: 55 [30 93] for ASD and 60 [36 87] for TD children). This analysis revealed a main effect of Electrodes further characterized by a Group by Electrodes interaction (F(2,68) = 10.53, *P* < 0.001, ${\upeta}_{\mathrm{p}}^2$ = 0.24) due to TD children presenting a larger P100 on Oz and O2 than O1. Again, the main effect of Group was no longer significant. When keeping only trials with a more homogeneous latency range, 78% of children with ASD and 78% of TD children presented a significant proportion of trials with a positive activity.

## Discussion

The present study aimed at investigating early visual responses in children with ASD. To this end, P100 amplitudes and latencies evoked by pattern-reversal checkerboards were measured in children with ASD and in TD peers.

Using an ERPs analysis, we report a smaller P100 amplitude to a pattern-reversal checkerboard stimulation in children with ASD, similar to that observed in adults^7^, in accordance with a vast literature showing atypical visual functioning in ASD^3–5,15,38^. Importantly, by controlling for ophthalmological issues that were present in an unexpectedly high number of ASD participants^39^, we are sure that our observations are not biased by the inclusion of participants with ophthalmological pathologies. Therefore, the current study suggests that these atypical sensory responses are not linked to ophthalmological pathologies and represent a genuine feature of ASD, present at all ages, rather than a compensatory mechanism observed only in adulthood consecutive to atypical visual experience.

ASD children differed from TD children regarding the lateralization of P100 component as an early hemispherical effect was found in TD children only: amplitude was larger in the right than in the left hemisphere. Previous studies have reported a lack of hemispherical difference in those with ASD^3,40^. Pei et al.^3^ found in the ASD group a selective amplitude reduction over the right hemisphere to steady-state VEPs pattern reversal. Abnormal hemispheric responses or lack of lateralization have also been described in the auditory domain^41–43^, suggesting that this effect is not visual-specific, but could be a core feature of ASD. A smaller activity to faces is often reported in the right hemisphere^44,45^; this hypo-activation is often discussed as an atypical social processing. As face perception is dominated by the right hemisphere^44,45^, an atypical face processing could be the consequence to this atypical lateralization of sensory processing.

To go further in our understanding of the reduced P100 amplitude in ASD, we performed two types of analyses on single trials as previously suggested for ASD^15,20,27,46^, but also for ADHD^22^. We measured inter-trial consistency^20^ by computing the proportion of trials showing a positive activity in the latency range of the P100 component. This method allows quantifying the consistency in the neural response recorded across trials and is more robust to inter-trial amplitude variation by categorizing trials in a binary fashion. ERPs are obtained by averaging many epochs with the underlying assumption that the response evoked by a stimulus is similar from trial-to-trial^36^. If this was the case, one would expect to observe 100% of positive trials in the latency range of the P100. It is noteworthy that no participant showed 100% of inter-trial consistency, although the proportion reached 90% in three TD children. In children with ASD, the maximum proportion observed was 79% highlighting an overall smaller inter-trial consistency in ASD. Inter-trial (in)consistency reflects variability that could be attributable to inter-trial desynchronization. Seventy-eight per cent of TD children presented a high inter-trial consistency in the P100 time window, but only 44% of ASD children presented a similar pattern. Therefore, 66% of children with ASD presented an inter-trial inconsistency possibly reflecting an inter-trial desynchronization. In the second analysis on single trials, we evaluated inter-trial latency variability of the st-P100, as measured by the MAD, and showed that the latency MAD was increased in children with ASD compared with controls^21^. Again, although 89% of TD children presented latency MAD values within 1 SD of the mean, only 44% of children with ASD presented a similar pattern.

Both approaches highlighted an heterogeneity in inter-trial variability in the autistic group: although a subgroup of autistic children (*n* = 6) presented patterns similar to the typically developing children, eight children presented altered responses regardless of the measure of inter-trial variability used; finally, a third group of ASD children (*n* = 4) presented atypical response in either measures (atypical inter-trial consistency: *n* = 2; atypical MAD value: *n* = 2). As this increased inter-trial variability may explain the amplitude reduction observed in the P100 measured on the ERPs, additional analyses were performed on reconstructed ERPs following the exclusion of trials with negative activity and trials with outlier latencies. Both controlled analyses abolished group differences in P100 amplitude, suggesting that the reduction in amplitude is solely driven by inter-trial variability. This important result demonstrates that amplitude reduction of the P100 is not the major difference between typical and autistic children, but is rather the consequence of an increased inter-trial variability in subgroups of children on the autism spectrum, consistent with what has been described in the auditory domain^20^. This increasing evidence of neural heterogeneity in ASD both at the individual and population level^20,21,47^ could explain the failure in identifying biomarkers that are both discriminant with regard to the typical population but also sensitive to variation of clinical symptoms within the spectrum. These results also further support the necessity of developing models that combine multiple clinical scores and neural profiles^20^ to better understand the complexity of the spectrum^48^.

The present study relates the reduced P100 amplitude to increased latency variability, suggesting that combined analyses of ERPs and single trials should be carried out when investigating visual responses in ASD across age. This increased variability in ASD is consistent with previous reports^15,20^ and supports the neural noise theory^26,27^. Enhanced response variability might be explained in turn by changes in attention^49–51^, but also by atypical ocular functioning^52–54^. As adults with ASD present abnormal and more heterogeneous fixation^8,55,56^, future studies should combine eye-tracking with EEG to explore the link between visual fixation and VEPs variability. The existence of subgroups in our sample of autistic children suggests that the neural response (in)consistency in terms of either amplitude or latency variability is not a characteristic of the entire autistic spectrum, but rather characterized specific children. The neural response inconsistency helps defining neural profiles which, when combined with other clinical markers provide a multidimensional functional assessment of each child. In turns, this would provide support for targeted interventions.

## References

[1] APA. *Diagnostic and Statistical Manual of Mental Disorders (DSM-5)* (American Psychiatric Association, Washington, DC, 2013).

[2] Mottron L (2007). Lateral glances toward moving stimuli among young children with autism: early regulation of locally oriented perception?. Dev. Psychopathol..

[3] Pei F, Baldassi S, Norcia AM (2014). Electrophysiological measures of low-level vision reveal spatial processing deficits and hemispheric asymmetry in autism spectrum disorder. J. Vis..

[4] Constable PA, Gaigg SB, Bowler DM, Thompson DA (2012). Motion and pattern cortical potentials in adults with high-functioning autism spectrum disorder. Doc. Ophthalmol..

[5] Milne E, Scope A, Pascalis O, Buckley D, Makeig S (2009). Independent component analysis reveals atypical electroencephalographic activity during visual perception in individuals with autism. Biol. Psychiatry.

[6] Weinger PM, Zemon V, Soorya L, Gordon J (2014). Low-contrast response deficits and increased neural noise in children with autism spectrum disorder. Neuropsychologia.

[7] Kovarski K (2016). Brief report: early VEPs to pattern-reversal in adolescents and adults with autism. J. Autism Dev. Disord..

[8] Frey HP, Molholm S, Lalor EC, Russo NN, Foxe JJ (2013). Atypical cortical representation of peripheral visual space in children with an autism spectrum disorder. Eur. J. Neurosci..

[9] Siper PM (2016). Rapid and objective assessment of neural function in autism spectrum disorder using transient visual evoked potentials. PLoS ONE.

[10] Anagnostou E, Taylor MJ (2011). Review of neuroimaging in autism spectrum disorders: what have we learned and where we go from here. Mol. Autism.

[11] Casanova MF (2006). Minicolumnar abnormalities in autism. Acta Neuropathol..

[12] Thye MD, Bednarz HM, Herringshaw AJ, Sartin EB, Kana RK (2017). The impact of atypical sensory processing on social impairments in autism spectrum disorder. Dev. Cogn. Neurosci..

[13] Robertson CE, Baron-Cohen S (2017). Sensory perception in autism. Nat. Rev. Neurosci..

[14] Odom JV (2010). ISCEV standard for clinical visual evoked potentials (2009 update). Doc. Ophthalmol..

[15] Milne E (2011). Increased intra-participant variability in children with autistic spectrum disorders: evidence from single-trial analysis of evoked EEG. Front Psychol..

[16] Brown AC, Chouinard PA, Crewther SG (2017). Vision research literature may not represent the full intellectual range of autism spectrum disorder. Front Hum. Neurosci..

[17] Chakrabarti B (2017). Commentary: critical considerations for studying low-functioning autism. J. Child Psychol. Psychiatry.

[18] Tager-Flusberg H, Kasari C (2013). Minimally verbal school-aged children with autism spectrum disorder: the neglected end of the spectrum. Autism Res..

[19] Dinstein I (2012). Unreliable evoked responses in autism. Neuron.

[20] Latinus M (2019). Atypical sound perception in ASD explained by inter-trial (in)consistency in EEG. Front Psychol..

[21] Milne Elizabeth, Gomez Rosanna, Giannadou Aikaterini, Jones Myles (2019). Atypical EEG in autism spectrum disorder: Comparing a dimensional and a categorical approach. Journal of Abnormal Psychology.

[22] Gonen-Yaacovi G (2016). Increased ongoing neural variability in ADHD. Cortex.

[23] Dinstein I (2010). Normal movement selectivity in autism. Neuron.

[24] Rubenstein JL, Merzenich MM (2003). Model of autism: increased ratio of excitation/inhibition in key neural systems. Genes Brain Behav..

[25] Simmons DR, Milne E (2015). Response to Davis and Plaisted-Grant: low or high endogenous neural noise in autism spectrum disorder?. Autism.

[26] Simmons DR (2009). Vision in autism spectrum disorders. Vis. Res..

[27] Dinstein I, Heeger DJ, Behrmann M (2015). Neural variability: friend or foe?. Trends Cogn. Sci..

[28] Butler JS, Molholm S, Andrade GN, Foxe JJ (2017). An examination of the neural unreliability thesis of autism. Cereb. Cortex.

[29] Livingston LA, Happé F (2017). Conceptualising compensation in neurodevelopmental disorders: reflections from autism spectrum disorder. Neurosci. Biobehav. Rev..

[30] APA. *Diagnostic and Statistical Manual of Mental Disorders (DSM-IV-TR)* (American Psychiatric Association: Washington, DC, 2000).

[31] Lord C (2000). The autism diagnostic observation schedule-generic: a standard measure of social and communication deficits associated with the spectrum of autism. J. Autism Dev. Disord..

[32] Lord C, Rutter M, Le Couteur A (1994). Autism Diagnostic Interview-Revised: a revised version of a diagnostic interview for caregivers of individuals with possible pervasive developmental disorders. J. Autism Dev. Disord..

[33] Schopler E, Reichler RJ, DeVellis RF, Daly K (1980). Toward objective classification of childhood autism: Childhood Autism Rating Scale (CARS). J. Autism Dev. Disord..

[34] Delorme A, Makeig S (2004). EEGLAB: an open source toolbox for analysis of single-trial EEG dynamics including independent component analysis. J. Neurosci. Methods.

[35] Aguera PE, Jerbi K, Caclin A, Bertrand O (2011). ELAN: a software package for analysis and visualization of MEG, EEG, and LFP signals. Comput. Intell. Neurosci..

[36] Ouyang G, Sommer W, Zhou C (2016). Reconstructing ERP amplitude effects after compensating for trial-to-trial latency jitter: a solution based on a novel application of residue iteration decomposition. Int. J. Psychophysiol..

[37] Wilcox R. *Introduction to robust estimation and hypothesis testing*. 3rd edn (Academic Press: Oxford, UK, 2011).

[38] Kornmeier J, Worner R, Riedel A, Bach M, Tebartz van Elst L (2014). A different view on the checkerboard? Alterations in early and late visually evoked EEG potentials in Asperger observers. PLoS ONE.

[39] Little Julie-Anne (2018). Vision in children with autism spectrum disorder: a critical review. Clinical and Experimental Optometry.

[40] Lazarev VV, Pontes A, deAzevedo LC (2009). EEG photic driving: right-hemisphere reactivity deficit in childhood autism. A pilot study. Int J. Psychophysiol..

[41] Orekhova EV (2012). Auditory cortex responses to clicks and sensory modulation difficulties in children with autism spectrum disorders (ASD). PLoS ONE.

[42] Orekhova EV (2009). The right hemisphere fails to respond to temporal novelty in autism: evidence from an ERP study. Clin. Neurophysiol..

[43] Charpentier J (2018). Emotional prosodic change detection in autism spectrum disorder: an electrophysiological investigation in children and adults. J. Neurodev. Disord..

[44] Puce A, Allison T, Asgari M, Gore JC, McCarthy G (1996). Differential sensitivity of human visual cortex to faces, letterstrings, and textures: a functional magnetic resonance imaging study. J. Neurosci..

[45] Kanwisher N, McDermott J, Chun MM (1997). The fusiform face area: a module in human extrastriate cortex specialized for face perception. J. Neurosci..

[46] David N (2016). Variability of cortical oscillation patterns: A possible endophenotype in autism spectrum disorders?. Neurosci. Biobehav Rev..

[47] Hahamy A, Behrmann M, Malach R (2015). The idiosyncratic brain: distortion of spontaneous connectivity patterns in autism spectrum disorder. Nat. Neurosci..

[48] Al‐Jawahiri Reem, Jones Myles, Milne Elizabeth (2019). Atypical neural variability in carriers of 16p11.2 copy number variants. Autism Research.

[49] Heinze HJ, Luck SJ, Mangun GR, Hillyard SA (1990). Visual event-related potentials index focused attention within bilateral stimulus arrays. I. Evidence for early selection. Electroencephalogr. Clin. Neurophysiol..

[50] Mangun GR, Hillyard SA (1990). Allocation of visual attention to spatial locations: tradeoff functions for event-related brain potentials and detection performance. Percept. Psychophys..

[51] Fontanini A, Katz DB (2008). Behavioral states, network states, and sensory response variability. J. Neurophysiol..

[52] Zhang B (2008). Effects of fixation instability on multifocal VEP (mfVEP) responses in amblyopes. J. Vis..

[53] Dalton KM (2005). Gaze fixation and the neural circuitry of face processing in autism. Nat. Neurosci..

[54] Perlman SB, Hudac CM, Pegors T, Minshew NJ, Pelphrey KA (2011). Experimental manipulation of face-evoked activity in the fusiform gyrus of individuals with autism. Soc. Neurosci..

[55] Johnson BP, Lum JA, Rinehart NJ, Fielding J (2016). Ocular motor disturbances in autism spectrum disorders: systematic review and comprehensive meta-analysis. Neurosci. Biobehav. Rev..

[56] Shirama A, Kanai C, Kato N, Kashino M (2016). Ocular fixation abnormality in patients with autism spectrum disorder. J. Autism Dev. Disord..

